# Association between parental education level and intelligence quotient of children referred to the mental healthcare system: a cross-sectional study in Poland

**DOI:** 10.1038/s41598-025-88591-3

**Published:** 2025-02-03

**Authors:** Urszula Sajewicz-Radtke, Ariadna Łada-Maśko, Michał Olech, Paweł Jurek, Łucja Bieleninik, Bartosz M. Radtke

**Affiliations:** 1 Laboratory of Psychological and Educational Tests, ul. Czarnieckiego 5A/1, Gdańsk, Poland; 2 Institute of Psychology, University of Gdansk, ul. Bażyńskiego 8, Gdańsk, 80-309 Poland; 3 Department of Psychology, Medical University of Gdansk, ul. M. Skłodowskiej-Curie 3a, Gdańsk, 80-210 Poland; 4 Institute of Pedagogy and Languages, University of Applied Sciences in Elbląg, ul. Czerniakowska 22, Elbląg, 82-300 Poland; 5 GAMUT – The Grieg Academy Music Therapy Research Centre, NORCE Norwegian Research Centre, Postboks 7800, Bergen, 5020 Norway

**Keywords:** Intelligence, IQ, Availability of mental healthcare, Parental educational level, Cognitive development, Counseling system, Psychology, Health care, Risk factors

## Abstract

Considering the gap in understanding of the link between parental education and child intelligence quotient (IQ), our study aimed to investigate the association between parental education and the IQ of children referred to the mental healthcare system, explore which parent’s education level is more influential, and examine the impact of the child’s age and sex on these relationships. This cross-sectional study included 80,303 children aged 3–18 years who were referred to the mental healthcare system between 2018 and 2023. We predefined IQ composite score (Full IQ Scale), as measured by the Stanford Binet 5 Intelligence Scale, Fifth Edition (SB-5) as the primary outcome; the remaining SB-5 composite scores were the secondary outcomes. Linear regression analysis was performed using staircase coding for ordinal predictors with several binary independent variables. A significant correlation was found between parental education levels and IQ of the sampled children, with higher levels of parents’ education predicting higher IQ scores, particularly with mother’s education explaining 18.23% of the variance in children’s overall intelligence. No significant interaction was observed between parental education and child’s sex in predicting child IQ. However, a significant interaction was observed with age, showing that IQ decreases with age in children of parents with lower education, while it increases with age in children of parents with higher education. Our study underscores the pivotal influence of parental education on the IQ levels of children referred to mental healthcare services. Maternal education level was a stronger predictor of child IQ, potentially because mothers tend to be the primary caregivers. These findings suggest the need for targeted support programs for caregivers, particularly those with lower education levels, to facilitate the early detection of developmental challenges. Integrated education and healthcare efforts are crucial for equitable mental healthcare access.

**Supplementary Information:**

The online version contains supplementary material available at 10.1038/s41598-025-88591-3.

## Introduction

The education levels of both mothers and fathers (“parental education”) play a crucial role in shaping children’s level of intelligence^1^. However, despite the positive correlation between intelligence and years of education, each can still serve as a moderator for the other in relation to life outcomes^2,3^. Parents with higher intelligence typically have intelligent children because of the high heritability of intelligence, starting at around 20% in childhood and escalating to approximately 70% in adolescence and 80% in adulthood^4^. However, genes do not explain all aspects of intelligence level. The most plausible explanation lies in the genotype-environment correlation, where slight genetic differences are amplified as children actively choose, adapt, and shape environments aligned with their genetic predispositions^5,6^. The impact of parental intelligence on home environment is evident, with the latter being positively correlated with children’s cognitive and psychomotor development. Notably, as the child’s age increases, so does the strength of this correlation, implying a continuous positive impact of the home-parenting environment on children’s school achievement, academic attainment, physical health, mental health, and cognitive abilities^4,7–9^. The home environment is shaped, among other factors, by family routines that mediate the association with the child’s school achievement, independent of the child’s intelligence^4^.

The relationship between parental education and access to mental health services has been generally understudied. Nevertheless, parents play a significant role as supporters and decision facilitators when seeking mental health support for youth. Parental education may influence at what point a child’s difficulties are deemed significant enough to warrant intervention from specialists, encompassing both educational and mental health domains^4^. Additionally, parents’ higher education levels are strongly associated with awareness of pediatric mental health problems. Health literacy significantly influences parents’ decisions to seek and utilize mental health services, aided by their awareness about facilitators and barriers to treatment access, and ensures equitable service use^10^.

In light of extensive research spanning several decades and the available data regarding the influence of parental education on child mortality rates^11^, there is an intriguing prospect of delving into the role of parental education in the domain of children’s mental health. Furthermore, the association between parental education and parents’ reports of their children’s mental health has been shown to be stronger than those related to income or social class^12^. Research also shows that higher levels of parental education may decrease the risk of depression in children and adolescents^13^. Additionally, studies conducted across various countries indicates that parents with higher levels of education tend to spend more time with their children compared to those with lower levels of education, further emphasizing the crucial role of parental involvement in shaping children’s well-being^14^. Thus, the present study aimed to investigate the association between parental education and the IQ of children referred to the mental healthcare system (Appendix A). Furthermore, we examined the associations between parental education and specific areas and factors related to children’s intelligence. Moreover, we examined which parent’s education levels were more influential in predicting the IQ of the sampled children. Additionally, we would like to check whether the child’s sex and age moderate the association between parental education and child IQ.

## Methods

### Study design and participants

We utilized cross-sectional individual-level data from 2018 to 2023 intelligence assessments using the Stanford Binet 5 Intelligence Scale, Fifth Edition (SB5), obtained via a Diagnostic Support System (DSS). The data from the DSS were provided by the Open Science Framework (OSF). The DSS also contains demographic data, including residence, child’s sex and age, and parental education. The analyzed data were derived from assessments of a total of *N* = 419,135 Polish children of both sexes aged 3;0–18;11 who had been referred by parents to the mental healthcare system. For the study, observations were specifically selected from this group of children for whom information regarding the following was available: full intelligence assessment and demographic data concerning parents’ education, the child’s age at the time of the study, and the child’s sex. The final sample consisted of *N* = 80,303 children, accounting for 19% of the original sample size.

No ethical approval was required for this study as it solely involved the analysis of preexisting secondary data with no primary data collection. The study adhered to the STROBE guidelines (Appendix B).

### Data measures

Parental education levels were classified according to the Polish Education System (see Appendix C). This qualitative ordinal variable was coded as 1 = primary or lower secondary, 2 = vocational, 3 = secondary, or 4 = higher. The analysis included the education level of mothers and fathers, and the variable encoding the highest education level among the parents.

The IQ of the children was determined the Polish version of the SB5 with Full IQ Scale, two areas (V IQ - Verbal IQ, NV IQ - Nonverbal IQ) and five factors (FR IQ - Fluid Reasoning IQ, KN IQ - Knowledge IQ, QR IQ - Quantitative Reasoning IQ, VS IQ - Visual-Spatial Processing IQ, WM IQ - Working Memory IQ)^15,16^. We predefined IQ composite score (Full IQ Scale), as measured by the SB5 as the primary outcome; the remaining SB-5 composite scores (V IQ, NV IQ, FR IQ, KN IQ, QR IQ, VS IQ, WM IQ ) were the secondary outcomes. In the analyses, all outcomes expressed on an IQ scale (*M* = 100; *SD* = 15 in the general population) were employed. The SB5 scores considered in the analyses are presented in Appendix D. Measurement of intelligence using the Polish version of the SB5 is characterized by very high reliability and good validity, as demonstrated in validation studies (see Appendix D)^17^.

### Statistical methods

The selected data for analysis comprised only complete observations concerning key variables (age, sex, intelligence, mother’s education, and father’s education), and no data imputation methods were applied. No data cleaning methods were utilized in this study owing to the stringent criteria for selecting observations for analysis. Before the analyses, technical validation was conducted, including checks of variable ranges and distributions, identification of result inconsistencies, and identification of outliers. However, data inspection did not identify any problematic observations. This is because data correctness verification occurred at the diagnostic application level (e.g., checking whether the entered raw results fell within the expected value range). While diagnostic errors cannot be entirely ruled out, this risk was minimized through training and diagnostic context (any concerning or inconsistent results were verified and potentially corrected promptly by the diagnostician).

### Statistical analysis

To address the primary outcome of whether parental education level was associated with the IQ of children in the mental healthcare system, we utilized a series of linear regression models. These models employed various IQs as dependent variables, with the predictor variable being parents’ education level (mother’s, father’s, or the maximum of both). We conducted a linear regression using staircase coding of ordinal predictors along with several binary independent variables^18^.

## Results

Comprehensive data on intelligence were collected from *N* = 80,303 children aged 3;0–18;11 receiving support from the mental healthcare system across Poland. For each of these children, information regarding the educational level of both parents was obtained – this criterion was mandatory for inclusion in the study sample. It is worth clarifying that in 53,783 cases (i.e., 66.98% of the total sample) both parents had the same level of education, while only in the case of 26,520 children (i.e., 33.02% of the total sample) was there an educational gap between parents. Among the latter group, in 19,365 cases (i.e., 73.02% of parents with an educational gap), mothers had a higher level of education than fathers. The ages of the children in the sample ranged from early childhood to adolescence (*M* = 10.13, *SD* = 3.31). The sample consisted of both male (*n* = 50,957; 63%) and female (*n* = 29,346; 37%) children. Table 1 provides more detailed data on the demographic characteristics of the study participants in the entire sample, categorized by the maximum education level of both parents.

**Table 1:** Composition of the sample based on parental education.

	Maximum parents’ educational level *N* (%)				Total *N* (%)
	Primary or lower secondary	Vocational	Secondary	Higher	
Total	5,010 (6)	18,623 (23)	25,640 (32)	31,030 (39)	80,303 (100)
Age group					
Pre-schoolers (3;00–6;11)	840 (17)	2,512 (13)	4,774 (19)	6,856 (22)	14,982 (19)
Early-school-age children (7;00–9;11)	1,484 (30)	5,322 (29)	7,383 (29)	8,578 (28)	22,767 (28)
Adolescents (10;00–15;11)	2,274 (45)	9,682 (52)	12,428 (48)	14,384 (46)	38,768 (48)
Late adolescents (16;00–18;11)	412 (8)	1,107 (6)	1,055 (4)	1,212 (4)	3,786 (5)
Sex					
Male	2,969 (59)	11,601 (62)	16,541 (65)	19,846 (64)	50,957 (63)
Female	2,041 (41)	7,022 (38)	9,099 (35)	11,184 (36)	29,346 (37)
Place of residence					
City	2,238 (45)	8,605 (46)	8,432 (33)	5,688 (18)	55,189 (69)
Countryside	2,768 (55)	9,984 (54)	17,159 (67)	25,278 (81)	24,963 (31)
Missing data	4 (< 1)	34 (< 1)	49 (< 1)	64 (< 1)	151 (< 1)
Level of Intelligence					
Moderate intellectual disability (IQ 35–54)	882 (18)	1,764 (9)	1,533 (6)	1,093 (4)	5,272 (7)
Mild intellectual disability (IQ 55–69)	1,503 (30)	4,240 (23)	3,107 (12)	1,610 (5)	10,460 (13)
Below-average intelligence (IQ 70–84)	1,693 (34)	6,625 (36)	6,991 (27)	4,515 (15)	29,824 (25)
Average intelligence (IQ 85–114)	923 (18)	5,912 (32)	13,489 (53)	20,954 (68)	41,278 (51)
Above-average intelligence (IQ > 114)	9 (< 1)	82 (< 1)	520 (2)	2,858 (9)	3,469 (4)

Note: the last column of the table labeled Total is the sum of the observations in each row.

Table 2 summarizes the regression coefficients of all fitted models for the primary and secondary outcomes. As seen in the table, all results demonstrate a significant association between parental education and IQ of children referred to the mental health care system. For instance, the mean overall IQ of children whose mothers have primary and lower secondary education is 71.99. In contrast, for mothers with vocational education, it is 5.43 IQ points higher (i.e., 77.42); for mothers with secondary education, it is an additional 7.55 IQ points higher (i.e., 84.97); and finally, for mothers with higher education, it is a further 9.53 IQ points higher (i.e., 94.50). To summarize the overall model fit, mother’s education level explains 18.23% of the variance in the child’s overall intelligence level. In cases where the analysis included the maximum level of parents’ education, regression models explained a comparable portion of the variance in children’s IQ compared to models with mothers’ education level as the predictor. However, they explained more variance compared to models in which the father’s education was the predictor.

**Table 2:** Linear regression models testing the relationship between parental education and children’s intelligence levels referred to the Mental Healthcare System.

Regression model	IQ	V IQ	NV IQ	FR IQ	KN IQ	QR IQ	VS IQ	WM IQ
	B(SE)	B(SE)	B(SE)	B(SE)	B(SE)	B(SE)	B(SE)	B(SE)
Mother’s education								
Intercept	71.99 (0.18)	72.6 (0.18)	75.11 (0.18)	76.69 (0.18)	77.62 (0.16)	75.38 (0.18)	76.47 (0.18)	76.56 (0.18)
D1	5.43 (0.22)	5.34 (0.22)	4.79 (0.22)	5.09 (0.22)	3.78 (0.20)	4.66 (0.22)	4.75 (0.21)	4.50 (0.22)
D2	7.55 (0.16)	7.25 (0.17)	6.87 (0.16)	6.69 (0.16)	6.36 (0.15)	6.76 (0.16)	6.03 (0.16)	5.78 (0.17)
D3	9.53 (0.14)	9.40 (0.15)	8.39 (0.14)	8.08 (0.14)	8.25 (0.13)	8.57 (0.14)	7.75( 0.14)	7.16 (0.14)
R^2^ (%)	18.23	17.39	15.72	14.53	16.09	15.37	13.71	11.51
Father’s education								
Intercept	73.14 (0.19)	73.79 (0.19)	76.07 (0.18)	77.64 (0.19)	78.63 (0.17)	76.32 (0.19)	77.39 (0.18)	77.56 (0.19)
D1	5.99 (0.22)	5.81 (0.22)	5.36 (0.21)	5.67 (0.21)	4.20 (0.19)	5.29 (0.21)	5.16 (0.21)	4.79 (0.21)
D2	7.97 (0.15)	7.63 (0.15)	7.27 (0.15)	6.93 (0.15)	6.74 (0.14)	7.11 (0.15)	6.49 (0.15)	6.09 (0.15)
D3	8.35 (0.16)	8.29 (0.16)	7.28 (0.15)	7.14 (0.16)	7.29 (0.14)	7.49 (0.16)	6.69 (0.15)	6.24 (0.16)
R^2^ (%)	16.46	15.63	14.25	13.14	14.57	13.89	12.40	10.29
Maximum parents’ education								
Intercept	70.06 (0.24)	70.74 (0.24)	73.36 (0.23)	74.97 (0.24)	76.38 (0.21)	73.63 (0.23)	74.75 (0.23)	74.88 (0.24)
D1	6.31 (0.27)	6.18 (0.27)	5.58 (0.26)	5.80 (0.27)	4.16 (0.24)	5.52 (0.26)	5.60 (0.26)	5.36 (0.27)
D2	7.99 (0.16)	7.66 (0.16)	7.28 (0.16)	7.16 (0.16)	6.68 (0.15)	7.10 (0.16)	6.39 (0.16)	6.14 (0.16)
D3	9.80 (0.14)	9.64 (0.14)	8.64 (0.14)	8.33 (0.14)	8.49 (0.13)	8.77 (0.14)	7.98 (0.14)	7.34 (0.14)
R^2^ (%)	18.43	17.52	15.97	14.73	16.23	15.51	13.91	11.64

*Notes. p* < 0.001, D1—difference between ‘primary and lover secondary’ and ‘vocational,’ D2—difference between ‘vocational’ and ‘secondary,’ D3—difference between ‘secondary’ and ‘higher’; IQ—Full Scale IQ, V IQ—Verbal IQ, NV IQ—Nonverbal IQ, FR IQ—Fluid Reasoning IQ, KN IQ—Knowledge IQ, QR IQ—Quantitative Reasoning IQ, VS IQ—Visual-Spatial Processing IQ, WM IQ—Working Memory IQ.

**Fig. 1:**
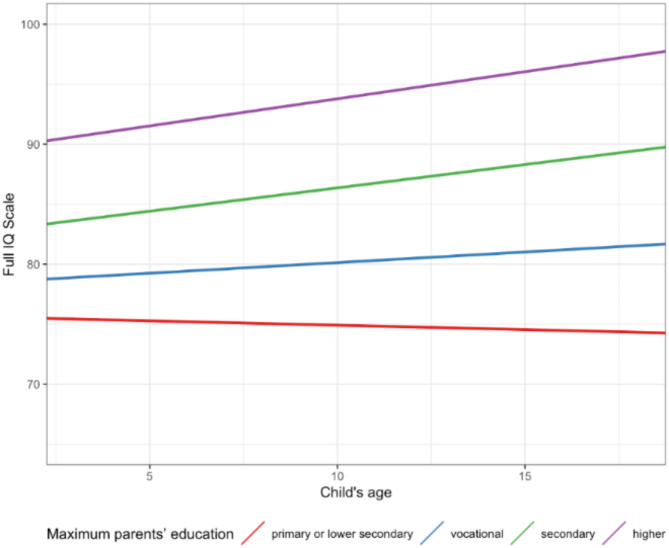
Interaction effects of parental education with child’s age on IQ in children referred to the mental healthcare system.

To examine whether parental education is associated with the IQ of children referred to the mental healthcare system depending on the child’s sex and age–we added interaction components with sex (Model A1) and age (Model A2) of children to the previously tested regression model (for the Full IQ Scale). Table 3 presents the results of these analyses. No significant interaction between parental education and child’s sex was noted in predicting their level of intelligence. Analyses of particular IQ areas (V IQ, NV IQ) and factors (FR IQ, KN IQ, QR IQ, VS IQ, WM IQ) are included in Appendix E. However, significant interactions with age were identified. To enhance the comprehension of this relationship, the results are elucidated in Fig. 1. The graphical representation illustrates a negative correlation between the age of children of parents with primary and lower secondary education referred to the mental healthcare system and their IQ. Conversely, the children of parents with higher education exhibited an inverse relationship, with IQ increasing with age. The analyses of specific IQ areas and factors are presented in Appendix E.

**Table 3:** Interaction effects of parental education with child’s sex and age on IQ in children referred to the Mental Healthcare System.

	Model A1 (with child’s sex as moderator)		Model A2 (with child’s age as moderator)	
	B	SE	B	SE
Intercept	70.47**	0.31	73.98**	0.75
D1	6.35**	0.34	4.31**	0.86
D2	7.80**	0.20	5.62**	0.54
D3	9.68**	0.18	7.92**	0.45
Child’s sex (female)	– 1.02*	0.48		
Child’s age			– 0.37**	0.07
D1 x child’s sex	– 0.21	0.54		
D2 x child’s sex	0.46	0.33		
D3 x child’s sex	0.34	0.30		
D1 x child’s age			0.19*	0.08
D2 x child’s age			0.23**	0.05
D3 x child’s age			0.19**	0.04
R^2^ (%)	18.47		18.57	

D1—difference between ‘primary and lover secondary’ and ‘vocational,’ D2—difference between ‘vocational’ and ‘secondary,’ D3—difference between ‘secondary’ and ‘higher’.

## Discussion

It is well known that parental education is crucial in shaping children’s IQ^1,2^. Our research provides for the first-time strong evidence of a correlation between parental education and the IQ of children referred to the mental healthcare system. Furthermore, the general IQ of the children explained the largest percentage of variance among all intelligence areas and factors examined. Parental education exhibited a stronger predictive capacity for a child’s verbal intelligence than for nonverbal intelligence. A detailed analysis of the structure of intelligence also indicated that parental education predominantly explained the level of the Knowledge factor (KN IQ) and explained the Working Memory factor (WM IQ). These results are consistent with the assumptions of the Cattel-Horn-Carroll theory of intelligence, indicating that verbal IQ is more dependent on environmental factors, whereas nonverbal IQ is more dependent on biological factors^19^. Studies have consistently shown that while genetic factors are significant in explaining the IQ range in children, other environmental and developmental influences undoubtedly also contribute significantly to shaping IQ^5,6,20^. Previous research also shows that parents’ level of education influences the shaping of the home environment, which is significant not only in the context of normative development but also in providing children mental healthcare^4^. The KN IQ is highly sensitive to environmental stimulation, encouraged both within formal education and the home environment. Children who have extensive educational experience and are surrounded by a stimulating home environment often have a greater chance of developing knowledge and skills, which can translate into their performance in this factor^19^. On the other hand, working memory is a process with strong neurobiological connotations^21^, hence parental education may have less explanatory power regarding a child’s performance in WM IQ.

Moreover, the results revealed that maternal education level was more influential in predicting general IQ, as well as specific intelligence areas and factors, among children referred to the mental healthcare system. Despite the steadily increasing trend of involved and nurturing fatherhood, women are still predominantly responsible for caring for children^22^. This seems to be a likely reason why maternal education is a stronger predictor of children’s IQ. Although mothers’ education explained a larger percentage of the variance (18.23%), it is important to remember that the percentage explained by fathers’ education was also high (16.46%). Therefore, collecting data on the education of both parents is important, as the higher education of either parent can be significant in organizing mental health support, especially in the context of evolving childcare trends.

Additionally, the child’s sex proved to be an insignificant factor in the relationship between parental education and children’s general IQ. However, further analysis of specific IQ areas and factors suggests that the child’s sex serves as a moderator in the relationship between parents’ educational level and the child’s KN IQ, indicating a stronger significance among boys. Previous studies have shown that parental education plays a significant role in children’s educational achievements^23^. However, our results provide important findings regarding whether parental education also matters in the context of referring children to the mental healthcare system, particularly when they need specialized assistance. It appears that sex is not a significant factor in this regard.

However, the age of the child was found to be a significant factor in predicting the IQ of children referred to the mental healthcare system based on parental education level. Our findings indicate a negative correlation between the age of children whose parents had primary and lower secondary education and their general IQ when referred to the mental healthcare system. In contrast, children of parents with higher education showed a positive correlation with general IQ, which increased with age.

To delve deeper into the interaction of parental education with the child’s age in the impact on the IQ of children referred to the mental healthcare system, we employed regression broken-line models^24^ for each of the four parental education levels. These models capture the piecewise linear relationship between the child’s age and general IQ previously established as a primary outcome, with three straight lines connected at two breakpoints reflecting the expected turning points in the Polish educational system (first breakpoint: transition between stage 1 and stage 2; second breakpoint: end of primary school exam; see Appendix C). Table E8 and Figure E1 in Appendix E present the results of the analysis. During the preschool period, as children grow older, they are brought to the mental healthcare system with progressively lower average IQ levels. However, between the ages of six and eight, this relationship reverses—older children brought to the mental healthcare system tend to have higher IQs. For parents with primary and lower secondary or vocational education, this trend begins shortly after the child starts school, and ends soon after the child completes primary education. Children’s school readiness is assessed in the sixth year of life, which is mandatory in kindergartens and schools. Given that the mental health care system is free and widely accessible, it is possible that, as a result of the assessment outcomes, parents make decisions to further diagnose their children’s school readiness, even if the child does not exhibit significant difficulties in cognitive functioning. However, primary education concludes with an external exam, the results of which significantly affect the selection of children’s future educational pathways. It is important to note that parents with primary and lower secondary or vocational education generally bring children with significantly lower levels of intellectual functioning to the mental healthcare system. For parents with secondary or higher education, the reversal of the initial trend lasts for a shorter duration (between ages 7 and 10 years) but it is more pronounced.

The study results highlight that ensuring equality in access to the mental healthcare system requires more than the provision of an open, universally accessible system. It is also crucial to identify at-risk groups using readily available data. Our results indicate that parental education level may be one such important indicator of risk.

Therefore, it seems beneficial to consider introducing educational programs for parents on normative child development and early signs of potential developmental difficulties. Although all parents should be included in these initiatives, caregivers with lower levels of education appear to require particular support. It is essential that these programs are grounded in evidence-based practices and consider socially sensitive factors such as low socioeconomic status, ethnicity, and disability^25^. We are aware of the significant financial and human resources required for widespread implementation of such programs. However, in the long run, it could enable faster identification of difficulties and reduce the costs associated with later assistance and treatment. A less direct solution could be expanding the scope of periodic medical screening programs to encompass more complex issues related to mental health and psychological aspects of child and adolescent development.

Our observations on the relationship between the education system and referrals to the mental healthcare system warrant further investigation. However, it seems essential to strengthen cooperation between the education sector and the healthcare system, as pivotal points in the education system, such as external examinations, may have a significant impact on the timing of seeking help for developmental challenges.

To the best of our knowledge, this is the first study to investigate the association between parental education level and IQ of children referred to the mental healthcare system. It is particularly noteworthy that data on the education levels of both parents from a large sample were analyzed. Additionally, data on the child’s IQ level were analyzed using SB5, a method that allows for the broadest assessment of intelligence structure^26^. In the present study, we analyzed free and widely accessible data from the mental health care system. This allowed for the inclusion of individuals with a lower education or SES. Therefore, the results can be generalized to a wider population of children referred to mental healthcare system.

The present study has certain limitations. A significant limitation was the necessity to limit the analysis to 80,303 of 419,135 participants. For clarification, we conducted an additional comparison of the demographic composition of the study sample with the original sample minus the study sample (see Appendix F, Table F1). As shown, both samples (the study sample with available parental education data and the sample with missing data) do not differ significantly in terms of gender, age group, place of residence, and, most importantly, level of intelligence. Additionally, we replicated the model fit for the regression analysis testing the relationship between mother’s education and children’s intelligence levels referred to the mental healthcare system using the expanded sample, independent of father’s education information availability (see Appendix F, Table F2). As can be seen, the results of this analysis are stable and comparable to the previous ones, even when applied to a sample more than twice the size (*N* = 203,690). Regardless of the results of the comparisons outlined above, the missing data reported should still be considered a limitation in generalizing the results to the population of children referred to the mental healthcare system.

Secondly, for formal reasons, we lacked knowledge of the specific diagnoses provided to each participant. The data collection system is restricted by data protection regulations, which prevent the identification of individual participants for medical history analysis. Additionally, this study only examined a very narrow part of the diagnostic process, specifically IQ measurement. It is not known what other diagnostic procedures were performed or what their results were. Future research should broaden the scope of this study to include other psychological variables and aspects of mental health. However, our research emphasizes the importance of identifying demographic factors that are relatively easy to obtain, do not incur additional storage and acquisition costs, and can serve as indicators for identifying risk groups for exclusion, even in open and widely accessible assistance systems. In the context of mental health in children and adolescents, a deeper focus on factors that may influence the formation of the family environment is warranted so that potential difficulties can be identified at very early stages of a child’s life.

In summary, our study revealed a correlation between parental education and the IQ of children referred to the mental healthcare system. General IQ explained the largest variance among the examined intelligence areas, with parental education being more predictive of verbal intelligence than of nonverbal intelligence. Maternal education level had a stronger impact on children’s IQ, possibly because of mothers’ predominant role in childcare. However, it is crucial to collect data on the educational levels of both parents because of the potential protective effects of having at least one parent with a higher level of education.

The Polish mental health care system is open, free, and widely accessible. However, our research indicates that parents with lower educational levels are more likely to refer children with lower IQ scores to this system. This suggests that universal access to the mental healthcare system does not guarantee equal care access. Social factors, such as the caregiver’s educational level, may allow access to this system. Therefore, regardless of the type of mental healthcare system, increasing efforts and funding for caregiver education concerning children’s and adolescents’ developmental trajectories as well as mental health are essential.

## Electronic supplementary material

Below is the link to the electronic supplementary material.

Supplementary Material 1

## Data Availability

Data supporting the findings of this study are openly available in the Open Science Framework (OSF) at https://osf.io/qatrz/?view_only=a9a4ed5c7dd74af0b338ca120b85b7bc. The repository includes database files in two formats (Excel, Rda) and a source code file in the R language to facilitate working with the data. Correspondence and requests for materials should be addressed to Bartosz M. Radtke.
