# Supplementary material for: Association between parental education level and intelligence quotient of children referred to the mental healthcare system: a cross-sectional study in Poland
Source: Sci Rep. 2025 Feb 3;15:4142. doi: 10.1038/s41598-025-88591-3 (PMC11790822; doi:10.1038/s41598-025-88591-3)
Supplement: Supplementary file 1 — Supplementary Material 1 [file 41598_2025_88591_MOESM1_ESM.pdf]

Supplementary Appendix for *Association between Parental Education Level and Intelligence Quotient of Children referred to the Mental Healthcare System: A Cross-sectional Study in Poland*

Urszula Sajewicz-Radtke, Ariadna Łada-Maśko, Michał Olech, Paweł Jurek, Łucja Bieleninik, Bartosz M. Radtke

Page

Appendix A: Description of Mental Healthcare System in Poland. 2

1. Mental health protection for children and adolescents in Poland - Care organized within the Ministry of Health 2-3
2. Psychological and educational assistance and counseling for children and adolescents in Poland - Care organized within the Ministry of Education 3-4

Appendix B: STROBE checklist for reporting of cross-sectional studies 5-6

Appendix C: Description of Polish Education System 7-8

Figure C1: Scheme of the Polish Education System 7

Table C1: Types of schools in Polish Education System 8

Appendix D: Description and characteristics of the Stanford-Binet Intelligence Scales, Fifth Edition 9

Table D1. Spearman-Brown split-half reliability coefficients for the 8 core scales of SB5 broken down by age groups 9

Table D2. Means, standard deviations, and correlations between SB5 IQ with WAIS- R(PL) IQ scores 9

Appendix E: Supplementary statistical analyses for particular IQ areas and factors 10-13

Table E1. Interaction Effects of Parents' Education Level with Child's Sex and Age on Verbal IQ in Children Referred to the Mental Healthcare System 10

Table E2. Interaction Effects of Parents' Education Level with Child's Sex and Age on Nonverbal IQ in Children Referred to the Mental Healthcare System 10

Table E3. Interaction Effects of Parents' Education Level with Child's Sex and Age on Fluid Reasoning IQ in Children Referred to the Mental Healthcare System 11

Table E4. Interaction Effects of Parents' Education Level with Child's Sex and Age on Knowledge IQ in Children Referred to the Mental Healthcare System 11

Table E5. Interaction Effects of Parents' Education Level with Child's Sex and Age on Quantitative Reasoning IQ in Children Referred to the Mental Healthcare System 12

Table E6. Interaction Effects of Parents' Education Level with Child's Sex and Age on Visual-Spatial Processing IQ in Children Referred to the Mental Healthcare System 12

Table E7. Interaction Effects of Parents' Education Level with Child's Sex and Age on Working Memory IQ in Children Referred to the Mental Healthcare System 13

Table E8. Results of Regression Models with Broken-Line Relationships for Interaction of Parents' Education Level with Child's Age on IQ in Children Referred to the Mental Healthcare System 13

Figure E1. Interaction Effects of Parents' Education Level with Child's Age on IQ in Children Referred to the Mental Healthcare System – Regression Models with Broken-Line Relationships 13

Appendix F: Supplementary statistical analyses for missing data 14

Table F1. Composition of the Sample compared to the Original Sample minus the Study Sample 14

Table F2. Linear Regression Models Testing the Relationship Between Mother's Education and Children's Intelligence Levels Referred to the Mental Healthcare System – Expanded Sample, Independent of Father's Education Information Availability (N = 203,690) 14

Appendix A: Description of the Mental Healthcare System in Poland.

Mental health support for children and adolescents in Poland is organized and supervised by both the Ministry of Health and the Ministry of Education. Each of these ministries has its own model of care, with no overlapping tasks, although the forms of support are often similar and the specialists working in them possess similar (sometimes identical) competencies and qualifications.

1. Mental health protection for children and adolescents in Poland - Care organized within the Ministry of Health

Legal acts:

- Ustawa z dnia 19 sierpnia 1994 r. o ochronie zdrowia psychicznego Dz. U. z 2017 r., poz. 882 https://dziennikustaw.gov.pl/D2017000088201.pdf
- Rozporządzenie Rady Ministrów z dnia 8 lutego 2017 r. w sprawie Narodowego Programu Ochrony Zdrowia Psychicznego na lata 2017–2022 https://dziennikustaw.gov.pl/D2017000045801.pdf
- Rozporządzenie Rady Ministrów z dnia 30 października 2023 r. w sprawie Narodowego Programu Ochrony Zdrowia Psychicznego na lata 2023-2030 https://isap.sejm.gov.pl/isap.nsf/download.xsp/WDU20230002480/O/D20232480.pdf

Mental health protection for children and adolescents is organized in facilities providing psychological and psychiatric services, at three levels of referral.

Legal basis: Regulation of the Minister of Health of August 14, 2019, amending the regulation on guaranteed benefits in the field of psychiatric care and addiction treatment.

Level I of Referral:

Center for Community Psychological and Psychotherapeutic Care for Children and Adolescents

- This facility provides assistance to children and adolescents up to 21 years of age who are attending secondary schools, until completion.
- Individuals under 18 years of age must have the consent of a legal guardian to use the services.
- Services at Level I center are accessible without the need for a referral from a doctor or any other institution.
- At the team or center, you can access the following services:
  - Diagnostic psychological consultation
  - Psychological consultation
  - Individual psychotherapy session
  - Family psychotherapy session
  - Group psychotherapy session
  - Psychosocial support session
  - Home or community visit/consultation
  - Visit by a person conducting community therapy
- Services are provided by a team of specialists: psychologists, psychotherapists, and social workers.
- These teams and centers are not intended for individuals requiring psychiatric diagnosis or pharmacotherapy, hence there are no psychiatrists on the teams.
- The first visit should be realized within 7 days of registration.
- The program emphasizes community interventions, with a requirement that at least 15% of services are conducted in the patients' homes.

Level II of Referral:

In situations where advice from a psychiatrist is needed, the patient should go to:

- Center for Mental Health for Children and Adolescents - child mental health clinic
  - Outpatient services are provided by psychiatrists, psychologists, and psychotherapists within the child mental health clinic.
- Center for Mental Health for Children and Adolescents
  - Assistance is provided within the child mental health clinic and on the daily psychiatric and rehabilitation wards for children.
  - Outpatient and daily services are provided by psychiatrists, psychologists, psychotherapists, and occupational therapists accordingly.
- Referral is not required for services provided at the Center for Mental Health for children and adolescents and within the child mental health clinic at level II of referral.
- The first visit should occur within 7 days of registration.

Level III of Referral:
Highly Specialized 24-Hour Psychiatric Care Center:

- In the psychiatric ward for children, inpatient services are provided by psychiatrists, psychologists, psychotherapists, and occupational therapists.
- Referral from a specialist doctor is required for services provided at level III of referral.
- In a life-threatening situation, one can seek assistance from a level III referral center (hospital) without a referral.
- The goal is to have at least one such center functioning in each province.

The list of level I, II, and III centers can be found here (last updated on March 7, 2024): https://www.nfz.gov.pl/gfx/nfz/userfiles/_public/dla_pacjenta/informacje_o_swiadczeniach/swiadczenia_-_opieka_transgranic/bks_i-iii_poziom_referencyjny_dane_do_publikacji_wg_stanu_07.03.2024.xlsx

2. Psychological and Educational Assistance and Counseling for Children and Adolescents in Poland - Care organized within the Ministry of Education

Legal Acts:

- Rozporządzenie Ministra Edukacji Narodowej z dnia 9 sierpnia 2017 r. w sprawie zasad organizacji i udzielania pomocy psychologiczno-pedagogicznej w publicznych przedszkolach, szkołach i placówkach https://isap.sejm.gov.pl/isap.nsf/download.xsp/WDU20170001591/O/D20171591.pdf
- Rozporządzenie Ministra Edukacji Narodowej z dnia 1 lutego 2013 r. w sprawie szczegółowych zasad działania publicznych poradni psychologiczno-pedagogicznych, w tym publicznych poradni specjalistycznych https://isap.sejm.gov.pl/isap.nsf/download.xsp/WDU20230002499/O/D20232499.p

Specialized care aimed at mental health protection is organized and provided in kindergartens, schools, institutions, and through the activities of psychological-pedagogical counseling centers, including specialized ones.

- Educational system units provide psychological and educational assistance and counseling to children, students, parents, and teachers.
- This assistance takes place in kindergartens, schools, and psychological-pedagogical counseling centers.
- The interventions are divided into psychological-educational assistance and psychological-educational counseling.

Psychological-educational assistance:

- Legal basis: Regulation of the Minister of National Education of August 9, 2017, regarding the principles of organization and provision of psychological-pedagogical assistance in public kindergartens, schools, and institutions.
- It is one of the fundamental forms of educational activity in kindergartens, schools, and institutions.
- It involves:
    - Recognizing individual developmental and educational needs, as well as psycho-physical capabilities of children and students, and environmental factors influencing their functioning in kindergartens, schools, and institutions
    - Meeting the developmental and educational needs of students
- Providing students with psychological-pedagogical assistance, tailored to identified needs, is the responsibility of the preschool/school director, who:
    - In agreement with the governing body, makes decisions regarding the employment of teachers and specialists performing tasks in the field of psychological-pedagogical assistance, especially psychologists, educators, speech therapists, and career advisors.
    - Determines the forms of providing this assistance, the period of its provision, and the number of hours during which individual forms will be implemented with a given child/student.

Psychological-pedagogical counseling:

- Legal basis: Regulation of the Minister of National Education of February 1, 2013, regarding the detailed rules of operation of public psychological-pedagogical counseling centers, including public specialized counseling centers.
- Public psychological-pedagogical counseling centers, including public specialized counseling centers, provide psychological-pedagogical assistance to children (from birth) and adolescents, as well as assistance in choosing educational paths and professions. They also provide psychological-pedagogical assistance to parents and teachers related to the upbringing and education of children

and adolescents, and support kindergartens, schools, and institutions in carrying out educational, developmental, and care tasks.

- Specialized counseling centers conduct activities aimed at specific, homogeneous problems, taking into account the needs of the local community.
- Running public psychological-pedagogical counseling centers is the responsibility of the educational authorities of districts, and the governing body of the counseling center determines its operating area.
- Assistance provided by public psychological-pedagogical counseling centers is voluntary and free of charge, upon the request of a parent or legal guardian.
- Tasks of public psychological-pedagogical counseling centers include:
    - Psychological and educational diagnosis of children and adolescents
    - Issuing opinions and decisions regarding the education and upbringing of children and adolescents
    - Providing direct assistance to students and parents
    - Implementing preventive and supportive tasks in supporting the educational and developmental function of kindergartens, schools, and institutions, including supporting teachers in solving educational and developmental problems
    - Supporting kindergartens, schools, and institutions
- Diagnosing at the counseling center, as well as issuing information about the results of the conducted diagnosis or opinion, is carried out at the request of a parent or adult student.
- The opinion issued by the counseling center is forwarded to the educational unit attended by the student only at the request of the parent or adult student.
- The aim of diagnosing a child is primarily to determine their individual developmental and educational needs, psycho-physical capabilities, to explain the mechanisms of their functioning in relation to the reported problem, and to indicate a way to solve this problem.
- The opinion from the counseling center is necessary for adjusting the external examination concluding subsequent stages of education.

Appendix B: STROBE checklist for reporting of cross-sectional studies

STROBE Statement—Checklist of items that should be included in reports of cross-sectional studies

	Item No	Recommendation	Page No
**Title and abstract**	1	(*a*) Indicate the study's design with a commonly used term in the title or the abstract	1-2
		(*b*) Provide in the abstract an informative and balanced summary of what was done and what was found	2
**Introduction**			
Background/rationale	2	Explain the scientific background and rationale for the investigation being reported	2
Objectives	3	State specific objectives, including any prespecified hypotheses	2-3
**Methods**			
Study design	4	Present key elements of study design early in the paper	3
Setting	5	Describe the setting, locations, and relevant dates, including periods of recruitment, exposure, follow-up, and data collection.	3
Participants	6	(*a*) Give the eligibility criteria, and the sources and methods of selection of participants.	3
Variables	7	Clearly define all outcomes, exposures, predictors, potential confounders, and effect modifiers. Give diagnostic criteria, if applicable.	3
Data sources/ measurement	8*	For each variable of interest, data sources and details of methods of assessment (measurement). Describe comparability of assessment methods if there is more than one group.	3
Bias	9	Describe efforts taken to address potential sources of bias.	7
Study size	10	Explain how the study size was arrived at.	3
Quantitative variables	11	Explain how quantitative variables were handled in the analyses. If applicable, describe which groupings were chosen and why.	3
Statistical methods	12	(*a*) Describe all statistical methods, including those used to control for confounding.	3
		(*b*) Describe any methods used to examine subgroups and interactions.	3
		(*c*) Explain how missing data were addressed	3
		(*d*) If applicable, describe analytical methods taking account of sampling strategy.	NA
		(*e*) Describe any sensitivity analyses.	NA
**Results**			
Participants	13*	(a) Report numbers of individuals at each stage of study—e.g. numbers potentially eligible, examined for eligibility, confirmed eligible, included in the study, completing follow-up, and analyzed.	3-4
		(b) Give reasons for non-participation at each stage.	NA
		(c) Consider use of a flow diagram.	NA
Descriptive data	14*	(a) Give characteristics of study participants (e.g. demographic, clinical, social) and information on exposures and potential confounders.	3-4
		(b) Indicate number of participants with missing data for each variable of interest.	4
Outcome data	15*	Report numbers of outcome events or summary measures.	NA
Main results	16	(*a*) Give unadjusted estimates and, if applicable, confounder-adjusted estimates and their precision (e.g. 95% confidence interval). Clarify which confounders were adjusted for and why they were included.	4-5
		(*b*) Report category boundaries when continuous variables were categorized.	NA
		(*c*) If relevant, consider translating estimates of relative risk into absolute risk for a meaningful time period.	NA
Other analyses	17	Report other analyses done—e.g. analyses of subgroups and interactions, and sensitivity analyses.	6-8
**Discussion**			
Key results	18	Summarize key results with reference to study objectives.	6
Limitations	19	Discuss limitations of the study, taking into account sources of potential bias or imprecision. Discuss both direction and magnitude of any potential bias.	8

Interpretation	20	Give a cautious overall interpretation of results considering objectives, limitations, multiplicity of analyses, results from similar studies, and other relevant evidence.	7-8
Generalisability	21	Discuss the generalizability (external validity) of the study results.	7-8
**Other information**			
Funding	22	Give the source of funding and the role of the funders for the present study and, if applicable, for the original study on which the present article is based.	8

*Give information separately for exposed and unexposed groups.

Appendix C: Description of Polish Education System

Figure C1: Scheme of the Polish Education System

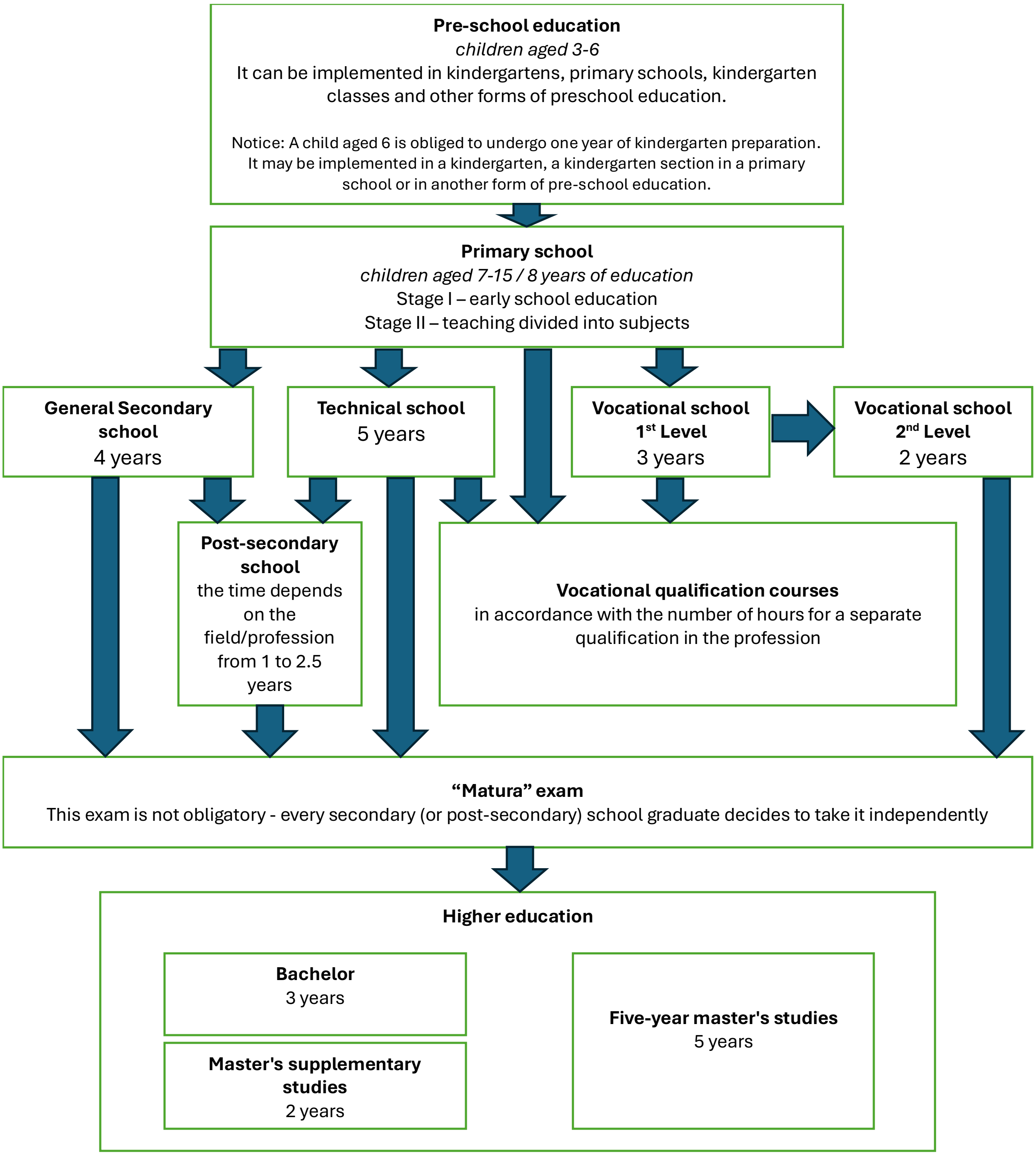

<u>School obligation:</u>

- Education is compulsory until the age of 18.
- A child's compulsory education begins at the beginning of the school year in the calendar year, in which the child turns seven and lasts until he or she completes primary school, but no longer than until he or she turns 18.

Table C1: Types of schools in Polish Education System

Type of school	Additional information
**Primary school**	In the eighth grade, there is an exam covering the following subjects: Polish, mathematics, and a foreign language.<br>The results obtained in the exam and the final grades in the primary school leaving certificate are the basis for studying at the next stage of secondary school education.
**General Secondary school**	Completion of general secondary school provides general secondary education, but does not provide a specific profession.
**Technical school**	During their studies, students of a technical secondary school take an external state examination leading to the title of technician. Completing a technical secondary school provides qualifications to perform a specific vocational education profession.
**Vocational school 1st Level**	After completing vocational training and passing the vocational or journeyman examination, the graduate will obtain a vocational diploma or journeyman certificate, as well as basic vocational education.<br>A graduate can take up work or continue education at a vocational school – 2nd Level or a general secondary school for adults.
**Vocational school 2nd Level**	Students take an external state examination conducted by the district examination commission. After successfully passing the vocational exam and graduating from school, a graduate of a second-cycle vocational school obtains the title of technician.

Special education:

- The basis for providing a child/student with special care and education is a decision affirming the need for special education issued by a public psychological and pedagogical counseling center.
- Special schools are organized for children and young people with a certificate of need for special education, including disabled people, with mild, moderate, or severe intellectual disabilities; those who are deaf and hard of hearing; those who are blind and visually impaired; and those with ASD.
- Education at special stage I vocational schools is provided to students with mild intellectual disabilities, who are socially maladjusted, and who are at risk of social maladjustment. Students with mild intellectual disabilities can also study at mainstream vocational schools.
- A student with a certificate of need for special education may be offered an extension of the education cycle, instead of repeating a grade. Depending on the educational stage, the learning period can be extended by one or two years.
- Education for the disabled, socially maladjusted, and at-risk of social maladjustment may be conducted until the end of the school year in the calendar year in which the student completes: 20 years of age in the case of primary school and/or 24 years of age in the case of secondary school.

Legal acts:

- Ustawa z dnia 14 grudnia 2016 r. Prawo oświatowe (Dz. U. z 2023 r. poz. 900, 1672, 1718 i 2005)
- Rozporządzenie Ministra Edukacji Narodowej z dnia 9 sierpnia 2017 r. w sprawie warunków organizowania kształcenia, wychowania i opieki dla dzieci i młodzieży niepełnosprawnych, niedostosowanych społecznie i zagrożonych niedostosowaniem społecznym (Dz. U. z 2020 r. poz. 1309)
- Study prepared by the Greater Poland Education Board (https://ko.poznan.pl/wp-content/uploads/2022/03/wielkopolski-informator_skrot_pl.pdf)

Appendix D: Description and characteristics of the Stanford-Binet Intelligence Scales, Fifth Edition

To measure intelligence and cognitive abilities, the main variables under study, the results of diagnoses using the Polish version of the Stanford Binet Intelligence Scales, Fifth Edition (SB5) (Roid, Sajewicz-Radtke, et al., 2017) were utilized. In the analyses, eight primary standard scores from the SB5 expressed on an IQ scale (M = 100; SD = 15 in the general population) were used. Standard scores were calculated using a diagnostic support application based on norm tables divided into detailed age groups derived from the data entered by the diagnostician. The following SB5 scores were considered in the analyses (Roid, 2003):

1. Full scale IQ, computed as the sum of all tasks in the SB5, encompassing subtests covering both verbal and nonverbal domains of cognitive ability. This provides a global summary of the current general level of intellectual functioning.
2. Verbal IQ, assessing intelligence through language-based tasks. It is calculated as the sum of all verbal tasks in the SB5, which typically involve vocabulary, comprehension, verbal reasoning, and verbal memory.
3. Nonverbal IQ, evaluating intelligence with the limited use of language. It is calculated as the sum of all nonverbal tasks in the SB5, which often include identifying patterns, completing visual sequences, and solving puzzles. Nonverbal IQ offers insight into an individual's cognitive abilities beyond language-based skills, providing a more comprehensive understanding of their overall intellectual functioning.
4. Fluid Reasoning IQ, measuring the ability to solve verbal and nonverbal problems using inductive or deductive reasoning.
5. Knowledge IQ, assessing the individual’s accumulation of general information acquired at home, school, or work.
6. Quantitative Reasoning IQ, evaluating the person’s ability to deal with numbers and numerical problem solving, whether with word problems or with pictorial relationships.
7. Visual-Spatial Processing IQ, measuring the ability to perceive patterns, relationships, special orientations, or the gestalt whole among diverse pieces of a visual display.
8. Working Memory IQ, assessing a class of memory processes in which diverse information stored in short-term memory is inspected, sorted, or transformed.

Measurement of intelligence and cognitive abilities using the Polish version of SB5 is characterized by very high reliability and good validity as demonstrated in validation studies (Roid, Jurek, et al., 2017). Tables D1 and D2 present results regarding the measurement quality in the Polish context.

Table D1· Spearman-Brown split-half reliability coefficients for the 8 core scales of SB5 broken down by age groups

SB5 scale	Age group						Mean
	2-3 (n = 442)	4-5 (n = 386)	6-8 (n = 371)	9-12 (n = 421)	13-16 (n = 403)	17-18 (n = 321)	
**Full Scale IQ**	0··99	0··98	0··97	0··97	0··98	0··97	0·98
**Verbal IQ**	0·98	0·96	0·95	0·95	0·96	0·95	0·96
**Nonverbal IQ**	0·97	0·95	0·94	0·95	0·95	0·93	0·95
**Fluid Reasoning IQ**	0·92	0·91	0·89	0·88	0·85	0·84	0·88
**Knowledge IQ**	0·95	0·91	0·87	0·90	0·93	0·92	0·91
**Quantitative Reasoning IQ**	0·96	0·90	0·88	0·88	0·91	0·89	0·90
**Visual-Spatial Processing IQ**	0·94	0·94	0·89	0·91	0·90	0·88	0·91
**Working Memory IQ**	0·95	0·88	0·91	0·90	0·90	0·90	0·91

Table D2· Means, standard deviations, and correlations between SB5 IQ with WAIS-R(PL) IQ scores

SB5 scale	WAIS-R		
	Overall IQ	Verbal IQ	Nonverbal IQ
**Full Scale IQ**	0·81**	0·68**	0·66**
**Verbal IQ**	0·83**	0·69**	0·68**
**Nonverbal IQ**	0·69**	0·55**	0·58**

N = 79; **p* < 0·01

Appendix E: Supplementary statistical analyses for particular IQ areas and factors

Table E1. Interaction Effects of Parents' Education Level with Child's Sex and Age on Verbal IQ in Children Referred to the Mental Healthcare System

	Model A1 (with child's sex as moderator)		Model A2 (with child's age as moderator)	
	B	SE	B	SE
Intercept	71·19**	0·31	72·63**	0·75
D1	6·23**	0·35	4·09**	0·86
D2	7·41**	0·20	5·61**	0·55
D3	9·47**	0·18	8·88**	0·45
child's sex (female)	-1·09*	0·48	–	–
child's age	–	–	-0·18**	0·07
D1 x child's sex	-0·21	0·55		
D2 x child's sex	0·63	0·34		
D3 x child's sex	0·47	0·30		
D1 x child's age			0·20**	0·08
D2 x child's age			0·20**	0·05
D3 x child's age			0·08	0·04

D1 – difference between 'primary and lower secondary' and 'vocational'; D2 - difference between 'vocational' and 'secondary'; D3 - difference between 'secondary' and 'higher'

Table E2. Interaction Effects of Parents' Education Level with Child's Sex and Age on Nonverbal IQ in Children Referred to the Mental Healthcare System

	Model A1 (with child's sex as moderator)		Model A2 (with child's age as moderator)	
	B	SE	B	SE
Intercept	73·68**	0·30	78·87**	0·73
D1	5·61**	0·34	3·88**	0·84
D2	7·19**	0·20	4·92**	0·53
D3	8·58**	0·17	5·84**	0·44
child's sex (female)	-0·80	0·47	–	–
child's age	–	–	-0·53**	0·07
D1 x child's sex	-0·17	0·53		
D2 x child's sex	0·20	0·33		
D3 x child's sex	0·16	0·29		
D1 x child's age			0·17*	0·08
D2 x child's age			0·21**	0·05
D3 x child's age			0·28**	0·04

D1 – difference between 'primary and lower secondary' and 'vocational'; D2 - difference between 'vocational' and 'secondary'; D3 - difference between 'secondary' and 'higher'

Table E3.Interaction Effects of Parents' Education Level with Child's Sex and Age on Fluid Reasoning IQ in Children Referred to the Mental Healthcare System

	Model A1 (with child's sex as moderator)		Model A2 (with child's age as moderator)	
	B	SE	B	SE
Intercept	74·77**	0·31	77·15**	0·75
D1	5·95**	0·34	3·35**	0·86
D2	6·98**	0·20	5·80**	0·54
D3	8·31**	0·18	7·50**	0·45
child's sex (female)	0·50	0·48	–	–
child's age	–	–	-0·21**	0·07
D1 x child's sex	-0·36	0·54		
D2 x child's sex	0·49	0·33		
D3 x child's sex	0·03	0·30		
D1 x child's age			0·23**	0·08
D2 x child's age			0·14**	0·05
D3 x child's age			0·09*	0·04

D1 – difference between 'primary and lower secondary' and 'vocational'; D2 - difference between 'vocational' and 'secondary'; D3 - difference between 'secondary' and 'higher'

Table E4. Interaction Effects of Parents' Education Level with Child's Sex and Age on Knowledge IQ in Children Referred to the Mental Healthcare System

	Model A1 (with child's sex as moderator)		Model A2 (with child's age as moderator)	
	B	SE	B	SE
Intercept	76·97**	0·28	87·97**	0·67
D1	4·03**	0·31	1·90**	0·77
D2	6·25**	0·18	2·22**	0·49
D3	8·22**	0·16	3·62**	0·40
child's sex (female)	-1·44**	0·44	–	–
child's age	–	–	-1·11**	0·06
D1 x child's sex	0·23	0·49		
D2 x child's sex	1·12**	0·30		
D3 x child's sex	0·75**	0·27		
D1 x child's age			0·23**	0·07
D2 x child's age			0·39**	0·04
D3 x child's age			0·48**	0·04

D1 – difference between 'primary and lower secondary' and 'vocational'; D2 - difference between 'vocational' and 'secondary'; D3 - difference between 'secondary' and 'higher'

Table E5. Interaction Effects of Parents' Education Level with Child's Sex and Age on Quantitative Reasoning IQ in Children Referred to the Mental Healthcare System

	Model A1 (with child's sex as moderator)		Model A2 (with child's age as moderator)	
	B	SE	B	SE
Intercept	74·43**	0·30	74·95**	0·74
D1	5·69**	0·34	4·76**	0·85
D2	7·04**	0·20	6·24**	0·54
D3	8·76**	0·17	7·67**	0·45
child's sex (female)	-1·96**	0·48	–	–
child's age	–	–	-0·13	0·07
D1 x child's sex	-0·61	0·54		
D2 x child's sex	0·04	0·33		
D3 x child's sex	0·06	0·29		
D1 x child's age			0·07	0·08
D2 x child's age			0·08	0·05
D3 x child's age			0·11**	0·04

D1 – difference between 'primary and lower secondary' and 'vocational'; D2 - difference between 'vocational' and 'secondary'; D3 - difference between 'secondary' and 'higher'

Table E6. Interaction Effects of Parents' Education Level with Child's Sex and Age on Visual-Spatial Processing IQ in Children Referred to the Mental Healthcare System

	Model A1 (with child's sex as moderator)		Model A2 (with child's age as moderator)	
	B	SE	B	SE
Intercept	75·52**	0·30	75·29**	0·72
D1	5·54**	0·33	5·03**	0·83
D2	6·36**	0·20	5·19**	0·52
D3	7·89**	0·17	7·52**	0·44
child's sex (female)	-1·90**	0·46	–	–
child's age	–	–	-0·05	0·07
D1 x child's sex	0·00	0·53		
D2 x child's sex	-0·04	0·32		
D3 x child's sex	0·30	0·28		
D1 x child's age			0·05	0·08
D2 x child's age			0·12**	0·05
D3 x child's age			0·05	0·04

D1 – difference between 'primary and lower secondary' and 'vocational'; D2 - difference between 'vocational' and 'secondary'; D3 - difference between 'secondary' and 'higher'

Table E7. Interaction Effects of Parents' Education Level with Child's Sex and Age on Working Memory IQ in Children Referred to the Mental Healthcare System

	Model A1 (with child's sex as moderator)		Model A2 (with child's age as moderator)	
	B	SE	B	SE
Intercept	74·61**	0·31	75·65**	0·75
D1	5·42**	0·35	2·71**	0·86
D2	6·05**	0·20	4·11**	0·54
D3	7·24**	0·18	6·79**	0·45
child's sex (female)	0·68	0·48	–	–
child's age	–	–	-0·07	0·07
D1 x child's sex	-0·10	0·55		
D2 x child's sex	0·28	0·34		
D3 x child's sex	0·26	0·30		
D1 x child's age			0·25**	0·08
D2 x child's age			0·21**	0·05
D3 x child's age			0·06	0·04

D1 – difference between 'primary and lower secondary' and 'vocational'; D2 - difference between 'vocational' and 'secondary'; D3 - difference between 'secondary' and 'higher'

Table E8. Results of Regression Models with Broken-Line Relationships for Interaction of Parents' Education Level with Child's Age on IQ in Children Referred to the Mental Healthcare System

Parents' Education Level	Slope1 (SE)	Slope 2 (SE)	Slope 3 (SE)	Break 1	Break 1 95% LCI	Break 1 95% UCI	Break 2	Break 2 95% LCI	Break 2 95% UCI
primary and lover secondary	-3·65 (0·94)	1·09 (0·13)	-5·07 (0·42)	6·01	5·43	6·60	13·79	13·42	14·16
vocational	-3·75 (0·84)	1·17 (0·07)	-4·70 (0·20)	5·50	5·09	5·90	13·46	13·27	13·64
secondary	-1·37 (0·17)	3·73 (0·24)	-1·67 (0·07)	7·61	7·40	7·82	10·27	10·12	10·42
higher	-1·05 (0·13)	3·29 (0·27)	-0·89 (0·07)	7·70	7·47	7·94	10·12	9·93	10·32

Figure E1. Interaction Effects of Parents' Education Level with Child's Age on IQ in Children Referred to Mental Healthcare System – Regression Models with Broken-Line Relationships

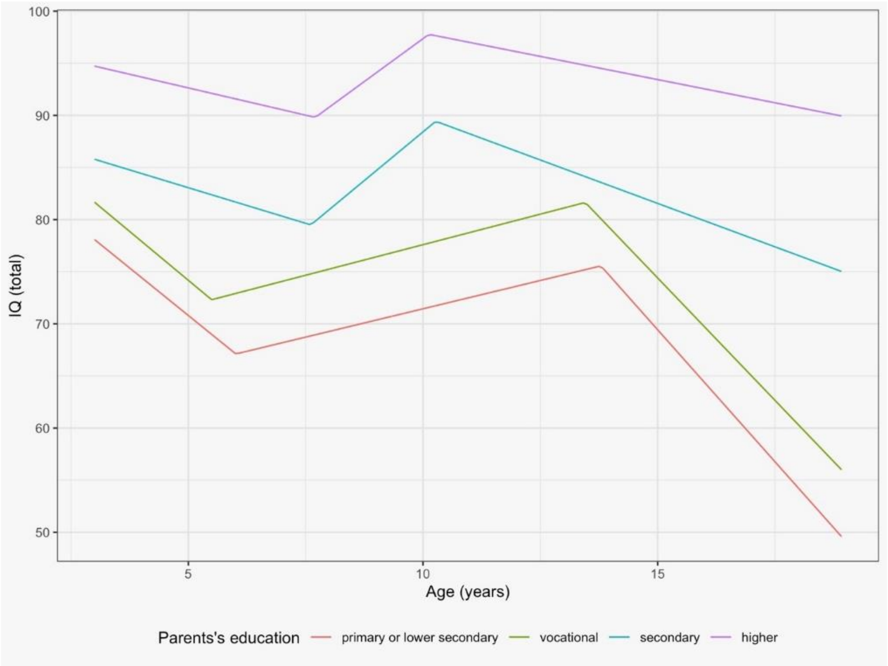

Appendix F: Supplementary statistical analyses for missing data.

Table F1. Composition of the Sample compared to the Original Sample minus the Study Sample

	Study sample (N = 80,303)		Original sample minus study sample (N = 338,832)	
	N	%	*N*	%
**Age group**				
Pre-schoolers (3;00–6;11)	14,982	19	65,161	19
Early-school-age children (7;00–9;11)	22,767	28	90,410	27
Adolescents (10;00–15;11)	38,768	48	165,601	49
Late adolescents (16;00–18;11)	3,786	5	17,660	5
**Sex**				
Male	50,957	63	210,648	62
Female	29,346	37	128,184	38
**Place of Residence**				
City	55,189	69	238,294	70
Countryside	24,963	31	97,110	29
Missing data	151	<1	3,428	1
**Level of Intelligence**				
Moderate intellectual disability (IQ 35–54)	5,272	7	20,023	6
Mild intellectual disability (IQ 55–69)	10,460	13	40,840	12
Below-average intelligence (IQ 70–84)	29,824	25	82,582	24
Average intelligence (IQ 85–114)	41,278	51	182,395	54
Above-average intelligence (IQ > 114)	3,469	4	12,992	4

Table F2. Linear Regression Models Testing the Relationship Between Mother's Education and Children's Intelligence Levels Referred to the Mental Healthcare System – Expanded Sample, Independent of Father's Education Information Availability (N = 203,690)

Regression Model	IQ	V IQ	NV IQ	FR IQ	KN IQ	QR IQ	VS IQ	WM IQ
	B(SE)	B(SE)	B(SE)	B(SE)	B(SE)	B(SE)	B(SE)	B(SE)
Intercept	72.65(0.12)	73.07(0.12)	75.87(0.11)	77.4(0.12)	78.37(0.1)	75.66(0.11)	77.08(0.11)	76.99(0.12)
D1	5.05(0.14)	4.8(0.14)	4.62(0.14)	4.73(0.14)	3.39(0.13)	4.48(0.14)	4.38(0.14)	4.19(0.14)
D2	7.89(0.11)	7.55(0.11)	7.19(0.1)	7.04(0.11)	6.53(0.1)	6.99(0.11)	6.39(0.1)	6.05(0.11)
D3	9.12(0.09)	9.08(0.09)	7.94(0.09)	7.69(0.09)	7.81(0.08)	8.22(0.09)	7.44(0.08)	6.95(0.09)
R^2 (%)	18.39	17.55	15.84	14.69	15.60	15.36	13.90	11.70
